# Emerging Neurological and Psychobiological Aspects of COVID-19 Infection

**DOI:** 10.3390/brainsci10110852

**Published:** 2020-11-12

**Authors:** Lyubka Tancheva, Maria Cristina Petralia, Simona Miteva, Stela Dragomanova, Ayten Solak, Reni Kalfin, Maria Lazarova, Dobri Yarkov, Rosella Ciurleo, Eugenio Cavalli, Alessia Bramanti, Ferdinando Nicoletti

**Affiliations:** 1 Department of Behavior Neurobiology, Institute of Neurobiology, Bulgarian Academy of Sciences, 1113 Sofia, Bulgaria; lyubkatancheva@gmail.com (L.T.); saalexandrova@gmail.com (S.M.); stela_dragomanova@abv.bg (S.D.); reni_kalfin@abv.bg (R.K.); m.lazarova@gmail.com (M.L.); 2 IRCCS Centro Neurolesi “Bonino-Pulejo”, Via Provinciale Palermo, Contrada Casazza, 98124 Messina, Italy; m.cristinapetralia@gmail.com (M.C.P.); rossella.ciurleo@irccsme.it (R.C.); abramanti@libero.it (A.B.); 3 Department of Pharmacology, Toxicology and Pharmacotherapy, Faculty of Pharmacy, Medical University, 9002 Varna, Bulgaria; 4 Institute of Cryobiology and food technologies, Agricultural Academy, 1407 Sofia, Bulgaria; aytensolak@abv.bg; 5 Faculty of Veterinary Medicine, Trakia University, 6000 Stara Zagora, Bulgaria; rector@uni-sz.bg; 6 Department of Biomedical and Biotechnological Sciences, University of Catania, Via S. Sofia 89, 95123 Catania, Italy; eugeniocavalli9@hotmail.it

**Keywords:** COVID-19, cytokines, central nervous system, Neuro-COVID, Parkinson’s disease, peripheral nervous system, SARS-CoV-2

## Abstract

The SARS-CoV-2 virus, first reported in December 2019 in China, is the causative agent of the current COVID-19 pandemic that, at the time of writing (1 November 2020) has infected almost 43 million people and caused the death of more than 1 million people. The spectrum of clinical manifestations observed during COVID-19 infection varies from asymptomatic to critical life-threatening clinical conditions. Emerging evidence shows that COVID-19 affects far more organs than just the respiratory system, including the heart, kidneys, blood vessels, liver, as well as the central nervous system (CNS) and the peripheral nervous system (PNS). It is also becoming clear that the neurological and psychological disturbances that occur during the acute phase of the infection may persist well beyond the recovery. The aim of this review is to propel further this emerging and relevant field of research related to the pathophysiology of neurological manifestation of COVID-19 infection (Neuro-COVID). We will summarize the PNS and CNS symptoms experienced by people with COVID-19 both during infection and in the recovery phase. Diagnostic and pharmacological findings in this field of study are strongly warranted to address the neurological and psychological symptoms of COVID-19.

## 1. Introduction

During the last two decades, novel viral epidemics have primarily affected the respiratory system. Among these, we can mention the Severe Acute Respiratory Syndrome (SARS) caused by coronavirus SARS-CoV, from 2002 to 2003, the swine flu caused by the H1N1 influenza virus in 2009 and the Middle East Respiratory Syndrome in 2012 caused by the Middle East respiratory syndrome virus (MERS)-CoV. More recently, the SARS-CoV-2 virus, first reported in December 2019 in China, caused the current pandemic of COVID-19 infection which at the time of writing (1 November 2020) has infected almost 43 million individuals and provoked the death of more than 1 million people [1].

The novel coronavirus SARS-CoV-2 challenges both the governments and healthcare systems worldwide with unprecedented social and economic distress. The global burden of the disease has motivated the scientific and medical community across the globe to focus their efforts on studying the pathogenetic mechanisms and the course of the disease, propelled by the urgent need to discover efficient prophylactic and therapeutic approaches.

The spectrum of clinical manifestations observed during COVID-19 infection varies from asymptomatic to critical life-threatening clinical conditions [1]. COVID-19 infection is classified into mild, moderate, severe, and critical depending on the symptoms. The asymptomatic or mild course is seen in some 80% of patients, another 15% experience a serious course requiring hospitalization and 5% have a critical illness [2,3]. These latter 5% of the patients may experience life-threatening conditions such as septic shock, cardiac and respiratory failures and multiple organ dysfunction [1]. According to the Johns Hopkins University statistics, the global death-to-case ratio is 4.5% (Johns Hopkins dashboard, 11 July 2020.

## 2. Multi-Organ Damages Related to COVID-19

The latest studies and emerging clinical data demonstrate that COVID-19 affects far more organs than just the respiratory system [4,5,6,7,8]. This is due to the strong affinity of SARS-CoV-2 for the human angiotensin-converting enzyme 2 (ACE2) receptor [9] expressed in several cell types including vascular endothelial cells, the renal tubular epithelium, and Leydig cells in the testes [10]. The ACE2 receptor plays a key role in the renin–angiotensin system regulating blood pressure, fluid and electrolyte balance and systemic vascular resistance [11]. The virus enters the host cells through binding with the ACE2 receptor. In contrast to its predecessor SARS-CoV that also recognizes the ACE2 as its receptor [12], the entry of SARS-CoV-2 to the host cell is further facilitated by another protein called furin that cracks the virus open and allows its genetic material to pour into the host cell. In return, the additional proteins required by the original SARS virus—pangolins—are only present in lung tissue which justifies their limitation to the respiratory system [13].

**Figure 1:**
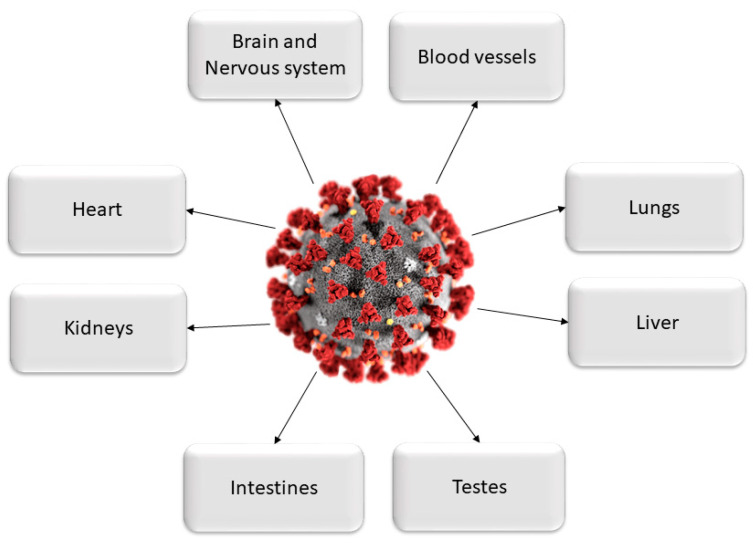
Major organs are affected by the damaging effects of COVID-19 infection.

**Figure 2:**
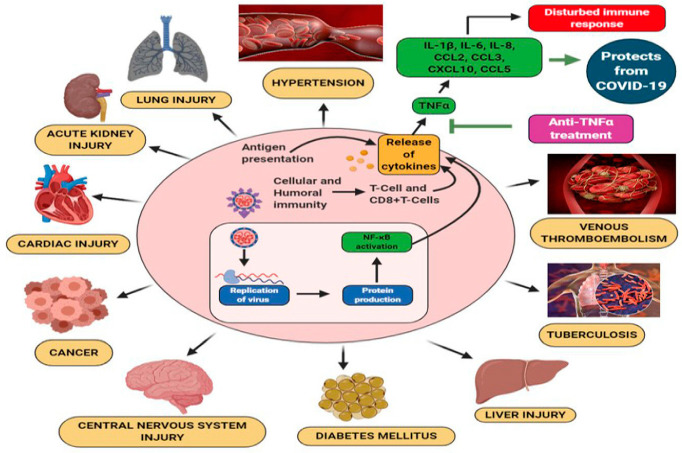
Coronaviruses pathogenesis, comorbidities and multi-organ damage [31].

Furin is present in all human cells and particularly in endothelial cells [14]. Following the viral entry into the human body, direct attack on organs and systems that are characterized by vast expressions of the ACE2 receptor is initiated. These organs include the heart, kidneys, blood vessels, liver, as well as the CNS. The resulting increase in several pro-inflammatory cytokines in response to infection [15] and their diffusion to other organs, may cause multi-organ damage. Clinical evidence for endothelial cell damage in the lungs, heart, kidneys, liver, and intestines in people with COVID-19 further establishes the disease as multi-organ and differentiates it from other known viral infections as influenza-like H1N1, SARS virus, which almost exclusively targets the lungs. Other viruses like Ebola or Dengue can also damage endothelial cells, but they are very different from viruses that typically infect the lungs [16]. This scenario is consistent with autopsies and biopsies that have reported SARS-CoV-2 viral particles not only in the nasal passages and throat, but also in the kidneys, liver, pancreas, and heart tissues as well as excreted in tears, stool and urine [6,17,18,19,20,21,22,23,24,25,26,27,28,29,30]. Hence, through this pathogenetic mechanism, SARS-CoV-2 acts more like a multi-organ disease, affecting the whole body rather than causing respiratory damage alone (Figure 1 and Figure 2) [8].

Recent evidence indicates that both the CNS and peripheral nervous system (PNS) could be targeted by the virus and that this could lead to clinical symptoms and associated diseases of different severity [32,33]. “Neuro-COVID” is the newly established term for the neurological, psychiatric and psychological clinical manifestations related to COVID-19.

In addition, there is also increasing awareness that the neurological and psychobiological disturbances caused by the virus during the acute phase of the infection may persist well beyond recovery. COVID-19 may provoke a broad spectrum of disorders that require strong efforts to dissect the pathogenetic mechanisms and identify therapeutic approaches facilitating the full functional recovery of the patients.

To address the growing epidemiological, socioeconomic and clinical relevance, the European Academy of Neurology (EAN) together with the Neurocritical Care Society (NCS) endorsed the Global Consortium Studies of Neurological Dysfunction in COVID-19 (GCS-Neuro-COVID)—an established formal collaboration which is the largest global network to date [34].

According to a recent Editorial in *The Lancet Neurology*, one of the priorities of this collaboration will be to develop consensus and data harmonization with uniform definitions [35].

The aim of the current review is to propel further this emerging field of research related to the pathophysiology of Neuro-COVID and consolidate a unitary vision some of the CNS and PNS symptoms and clinical manifestations observed during the infection and through recovery.

The pathogenetic mechanisms of COVID-19 and the clinical course of the disease are outside the focus of this review have been described in recent reviews by ourselves and others [36,37]. It may nonetheless be useful to emphasize the converging knowledge showing pleiotropic and still interconnected mechanisms of COVID-19 manifesting through enhanced inflammatory responses of the innate immune system and the inflammasome with the induction of a cytokine storm and the related or unrelated activation of thrombotic diathesis that may lead to diffuse intravascular coagulation (DIC) [15,36,37].

It is also known, but still useful to recall, the increased risk of severe COVID-19 posed by comorbidities such as obesity, type 2 diabetes and aging. In the elderly, these conditions may be linked by a process of chronic inflammation called “inflammaging” making them more prone to uncontrolled immune–inflammatory responses to the SARS-CoV-2 infection [38,39,40,41,42,43].

## 3. Pathogenesis of Neuro-COVID

### 3.1. Neurotropism of COVID-19

**Figure 3:**
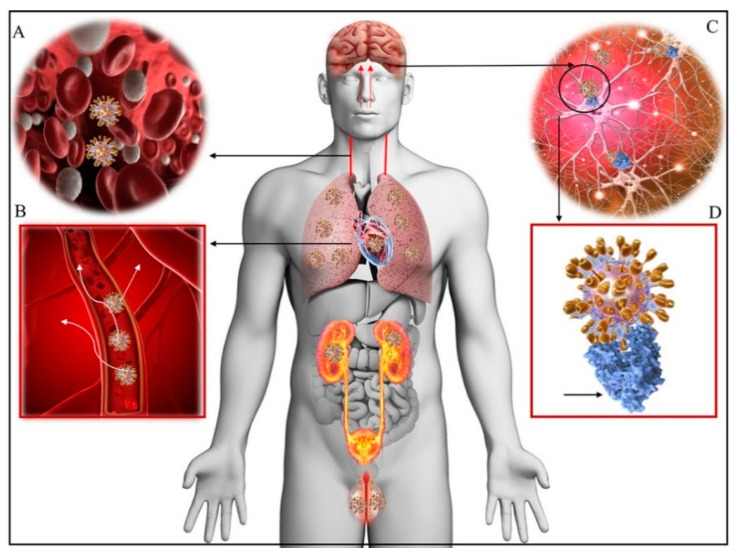
SARS-CoV-2 distribution in human body. **A**—Viremia: SARS-SoV-2 virus dissemination throughout the body via the bloodstream. **B**—Neurotropism via circulation and/or an upper nasal transcribrial route that enables the COVID-19 to modify the function of the blood–brain barrier or directly reach the brain. **C**—Virus binding and engaging with the ACE2 receptors. **D**—SARS-CoV-2 docks on the ACE2 via spike protein (blue); major organs–lungs, heart, kidneys, intestines, brain, and testicles, that express ACE2 receptors and are possible targets of COVID-19 (D, golden spikes) [45].

SARS-CoV-2 [44] exhibits neurotropism through its affinity for the ACE2 receptor in endothelial cells [45]. ACE2 is also found in the brain [45] and it is expressed in neural tissue (glial cells and neurons) making them a potential target for the virus [46]. The neurotropism of COVID-19 accords with the wide spectrum of neurological, psychiatric and psychological symptoms and syndromes affecting the entire nervous system in the course of infection (Figure 3).

However, it is still debated whether or not the virus directly infects the CNS. One study in this area of research did not report evidence of the presence of SARS-CoV-2 or its particles in the brain during the autopsy of patients with COVID-19, in spite of the viral invasion in various organs However, a first study did not report evidence of the presence of SARS-CoV-2 or its particles in the brain at autopsy of patients with COVID-19 infection in spite of the invasion of the virus in various organs such as lungs, liver and heart [47]. On the other hand, Moriguchi et al. [48] observed viral RNA in the cerebrospinal fluid of a patient with aseptic encephalitis and Paniz-Mandolfi et al. [49] reported the presence of SARS-CoV-2 viral particles in the neuronal and capillary endothelial cells in the frontal lobe tissue in a postmortem study. It has therefore been suggested that SARS-CoV-2 neuroinvasion, neuroinflammation and impairment of the blood–brain barrier (BBB) are responsible for the neurological symptoms [50].

### 3.2. Neuroinvasive and Neurotoxic Properties of Coronaviruses

Other members of the coronaviruses family have been reported to exhibit neuroinvasiveness and neurotoxicity that are clinically characterized by a broad spectrum of symptoms that include altered mental status, meningoencephalitis and seizures, as well as post-recovery complications such as headaches and memory loss [51]. It is also known that various neurological diseases may accompany the coronaviruses infections, such as encephalitis [52] optic neuritis [53], multiple sclerosis [54] and Parkinson’s disease (PD) [55].

In particular, the SARS-CoV epidemic from 2003 was associated with polyneuropathy, encephalitis, and ischemic stroke [56]. Cases of cerebral edema and meningeal vasodilation, infiltration of immune cells into the vascular wall, accompanied by the demyelination of nerve fibers and ischemic neuronal damage were also reported [57].

The MERS-CoV infection from 2012 also exhibited neurotoxic potential with symptoms that included neurological and neuropsychological impairments (25% of patients) and seizures (8.6%) [58]. Other neurological complications manifested as impaired consciousness, ischemic stroke, Guillain–Barré syndrome (GBS), and other neuropathies [59].

## 4. Direct and Indirect Mechanisms of SARS-CoV-2 CNS Invasion

It is generally accepted that coronaviruses may access the CNS through the hematogenous route and/or the neuronal retrograde dissemination.

The hematogenous route may occur via infected leukocytes that cross the BBB carrying the virus to the CNS and/or by the direct infection of brain microvascular endothelial cells, which express ACE2. Nonetheless, the hematogenous route does not seem to be involved in the CNS invasion by SARS-CoV-2, since, as discussed, virtually no viral particles were detected in non-neuronal cells of the infected brain areas in the early stage of infection [60].

**Figure 4:**
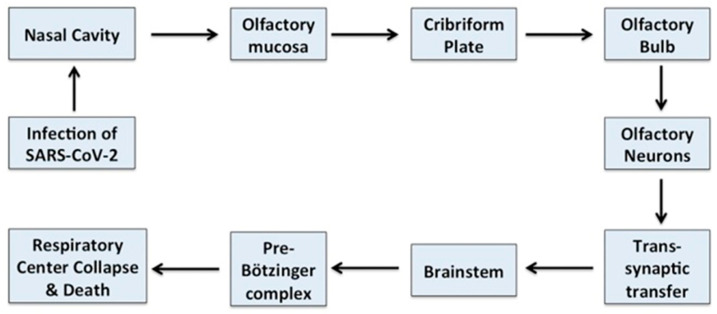
Schematic representation showing how SARSars-CoV-2 may infect the respiratory center of the brain. SARSars-CoV-2 may enter the brain through the olfactory mucosa present in the upper nasal cavity. From there, through olfactory axons, it may access the cribriform plate and project to the olfactory epithelium and olfactory bulb. SARSars-CoV-2 further migrates to deeper parts of the brain such as the thalamus and brainstem by trans-synaptic migration and targets the pre-Bötzinger complex, thus possibly causing the collapse of the respiratory center of the brain [62].

The neuronal route can occur via olfactory nerves and/or via the enteric nervous system (Figure 4). However, the fact that ACE2 in neurons in the olfactory system do not express ACE2 questions this route for SARS-CoV-2 entry into CNS. Hence, ACE2-independent mechanisms have been proposed to explain the entry of SARS-CoV-2 into host cells at least via the olfactory route. In contrast, ACE2 expression occurs in small intestine endothelial cells which connect with neurons in the enteric nervous system. This is consistent with the frequent occurrence of, gastrointestinal symptoms in a subset of patients with COVID-19 as well as with SARS-CoV-2 isolation from oral and anal swabs of these patients. In this way, SARS-CoV-2 could access the enteric nervous system, via the vagus nerve, as a possible pathway for the CNS [60]. In addition, recent studies have suggested that the transmembrane receptor neuropilin-1 may also be implicated in the neurotropism of COVID-19. In fact, NRPI is highly expressed in the respiratory and olfactory epithelium and and may enhance the entry of SARS-CoV-2 into the brain through this route. Recently NRP1 has also been shown to be expressed in the CNS, including olfactory-related regions such as the olfactory tubercles and para-olfactory gyri therefore strenghtering its possible role as additional site of entry for SARS-CoV-2 in the brain [61]. We will briefly depict below the proposed ACE2 receptor-dependent and ACE2-receptor-independent pathways of brain infection that may be used from SARS-CoV-2.

### 4.1. ACE2 Receptor-Dependent Pathway of CNS Invasion

The mechanism of SARS-CoV-2 cell entry through binding to its specific host receptor ACE2 was already discussed [12]. The ACE2 receptor is widely expressed in the brain stem and in the regions responsible for the regulation of cardiovascular function including the subfornical organ, paraventricular nucleus, and the nucleus of the tractus solitarius as well as rostral ventrolateral medulla. ACE2 has also been detected in glial cells and neurons.

A relevant finding is also that the nicotine stimulation of the nACh receptor can increase ACE2 expression in neural cells, which places smokers at a higher risk for neurological complications by SARS-CoV-2 [63].

The presence of SARS-Cov-2 in neurons and capillary endothelium cells in the postmortem examination of frontal lobe tissue supports the hematogenous pathway of viral entry into the brain [49,64].

After entering the general circulation, the virus may migrate to the brain where its replication begins and provokes a powerful immune response that, if uncontrolled, may lead to cerebral edema [45]. The combination of infection and immune response forms the typical clinical manifestation of viral meningitis/encephalitis.

### 4.2. ACE2 Receptor-Independent CNS Invasion

In a manner similar to both severe acute respiratory syndrome virus (SARS) and Middle East respiratory syndrome virus (MERS), SARS-CoV-2 might also take a direct trans-synaptic route via the olfactory bulb upon inhalation without using the ACE2 receptors. Animal studies demonstrated the potential of SARS-CoV to cause neuronal death by accessing the brain through the olfactory epithelium [65]. Virus invasion causes reactive astrogliosis and triggers a massive neuroinflammatory cascade in the CNS.

Recently, Takeshi et al. reported the first case of meningitis/encephalitis associated with SARS-CoV-2. They detected SARS-CoV-2 RNA in the cerebrospinal fluid (CSF) specimen [19], providing direct evidence of the neuroinvasiveness of the virus. This finding concurs with the hypothesis that SARS-CoV-2 may directly enter into the nervous system and that the clinical symptoms it induces could be directly caused by the virus and are not necessary a secondary effect of the immune response to the virus [48,66].

The gustatory or olfactory disorders observed in a significant proportion (33.9%) of COVID-19 patients are consistent with the hypothesis that the virus reaches the brain through neurons of the olfactory bulb by a direct trans-synaptic pathway [65].

## 5. Direct and Indirect Mechanisms of SARS-CoV-2 Neurotoxicity

Laboratory studies reveal increased inflammatory markers (white blood cell (WBC), neutrophils, C-reactive protein (CRP), and d-dimer) and evidence of multi-organ involvement as witnessed by elevated liver enzymes and abnormal renal function tests [67,68].

**Figure 5:**
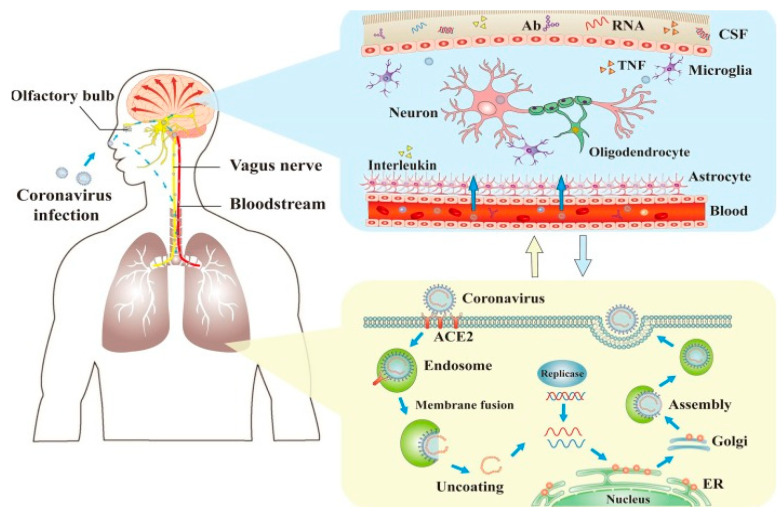
Mechanisms of coronaviral infection of the brain. SARS-CoV-2 can enter the nervous system directly through the olfactory nerve, and also through blood circulation and neuronal pathways, resulting in neurological disorders. Ab: antibody; ACE2: angiotensin-converting enzyme 2; CSF: cerebrospinal fluid; ER: endoplasmic reticulum; TNF: tumor necrosis factor. [72].

While some authors believe that neurological symptoms may be a consequence of the multi-organ damage caused by COVID-19 [68], it was also suggested that SARS-CoV-2 is capable of infecting the neurons. Supportive evidence comes from studies on so-called organoids that are small structures of brain tissue composed of human pluripotent stem cells manipulated to differentiate into neurons. Researchers demonstrate that SARS-CoV-2 infects the organoids and leads to partial neural death as well as a reduction of synapse formation [69,70]. Direct damage can occur when the virus enters the nervous system via the neuronal retrograde (olfactory bulb and/or n. Trigeminus), blood (overcoming BBB) and immune-mediated pathways (via immune cells that transmit the pathogen) Figure 5.

On the other hand, emerging evidence also indicates that in addition to direct viral invasion to the CNS, indirect CNS involvement through viral-mediated immune response is also possible [71].

After entering the central circulation, the viral particles bind to BBB endothelial cells and the brain–cerebrospinal fluid barrier epithelial cells that express ACE2 receptors [72].

Indirect mechanisms of neurotoxicity may be mediated by immunocompetent and inflammatory cells responsible for exacerbating the pro-inflammatory status of the CNS [73]. The increased activity and expression of immune cells, cytokines, radicals and other substances are associated with the most severe clinical cases with concomitant manifestations of neurological complications. The frequent cases of depression observed during COVID-19 infection may also be due to the massive release of pro-inflammatory cytokines that are implicated in the pathogenesis of this disease [74,75,76].

In support of these pathogenetic mechanisms, elevated levels of pro-inflammatory cytokines have been described after infection with COVID-19 including—IL-6, IL-12, IL-15, TNF-α [77] and interferon γ-induced protein 10 (IP-10, a T-cell chemoattractant chemokine) [78]. Elevated levels of IP-10 have also been linked to virus-induced demyelination [79].

Virus-induced hypoxia is another non-specific mechanism of damage targeting multiple organs and systems, including the nervous system. This indirect pathogenetic mechanism leads to pulmonary complications and damage to gas exchange [72] with subsequent hypoxia and metabolic acidosis, causing the vasodilation and edema of the brain. The condition is associated with a high risk of increased intracranial pressure and acute cerebrovascular accidents

The described direct and indirect CNS-damaging mechanisms of COVID-19 may be manifested in different sequences or occur simultaneously. It has, however, been proposed that neurological symptoms may precede respiratory ones [80].

Neuroinflammation may represent an important pathogenetic mediator by at least two mechanisms entailing the BBB damage induction of hypoxia and homeostatic dysregulation.

## 6. Neuroinflammation and Compromising the Blood–Brain Barrier

The systemic SARS-CoV-2-associated neuroinflammation compromises the BBB with consequential disturbance of brain homeostasis and the potential induction of neuronal death [67,81,82]. Although the endothelial cells lining the brain capillaries and comprising the first layer of BBB [83] differ in several aspects from the endothelial cells in the other parts of the body they are also a target for SARS-CoV-2 and become infected during viremia. This immune reaction involving cytokines compromises the function of the barrier [81]. Neural damage may lead to complications, systemic homeostatic dysregulation, multiple organ failure and acute respiratory failure that are the causes most closely linked to COVID-19 mortality [84].

Adding important insights into possible pathogenetic mechanisms at the base of Neuro-COVID are recent observations by Buzhdygan and coworkers [82]. By using postmortem brain tissue these authors have first confirmed that the ACE2 is ubiquitously expressed throughout various vessel calibers in the frontal cortex. Moreover, they also observed upregulated ACE2 expression in cases of hypertension and dementia. ACE2 was also detectable in primary human brain microvascular endothelial cells (hBMVEC) “in vitro”. They demonstrated that neither the S1, S2 nor a truncated form of the S1 containing only the receptor-binding domain impaired hBMVEC viability. The introduction of spike proteins in in vitro models of the BBB showed significant changes to barrier properties. In particular, S1 provoked the loss of barrier integrity in an advanced 3D microfluidic model of the human BBB. These findings accord with the possibility that SARS-CoV-2 spike proteins may evoke a pro-inflammatory response in brain endothelial cells that may contribute to a malfunctioning of the BBB. This scenario offers an additional pathogenetic mechanism by which SARS-CoV-2 may be responsible for the neurological consequences observed in COVID-19 patients [82].

### Neuroinflammation, Hypoxia and Homeostatic Dysregulation

In the brain, ACE2 receptors are predominantly present in the brain stem and the regions responsible for the regulation of cardiovascular function [85] which makes this CNS region more vulnerable to the damaging effect of SARS-CoV-2. This is why some authors hypothesize that CNS respiratory dysregulation may be the root cause for the respiratory failure observed in COVID-19 which is especially valid for patients without comorbidities and other risk factors [86].

Normal homeostasis is dependent on the adequate functioning of afferent and efferent sections of the autonomous nervous system. Damage to this function could lead to ventilatory function impairment as well as the exacerbation of the respiratory failure resulting in profound hypoxia. The combination of hypoxia with existent neuroinflammation leads to damage in the hippocampal and cortical areas manifesting as the neuropsychiatric effects of the virus [44,87].

In agreement, it has been suggested that a large-scale cerebral involvement may cause cerebral edema that could be lethal well before the homeostatic dysregulation is fully activated [45].

## 7. Neurological Complications Related to COVID-19

### Neurological Symptoms during COVID-19

Regardless of the precise mechanisms by which SARS-CoV-2 enters the CNS and how precisely it exerts its pathogenetic effects, a large variety of neuropsychiatric symptoms affecting both the CNS and the PNS are reported in patients with COVID-19 [77].

Nearly one-third of affected patients developed neurological symptoms that can either be related to the direct neurotoxicity of the virus or could be a complication of an existing disease. In both cases, this could lead to long-term damage [68].

Paterson and coworkers [88] have warned of a potential wave of COVID-19-linked brain damage as new evidence suggests that COVID-19 it can lead to severe neurological complications. The wide spectrum of neurological symptoms varies in severity and manifestation over time and is also dependent on variables like age comorbidity, sex, as well as geographical location.

**Figure 6:**
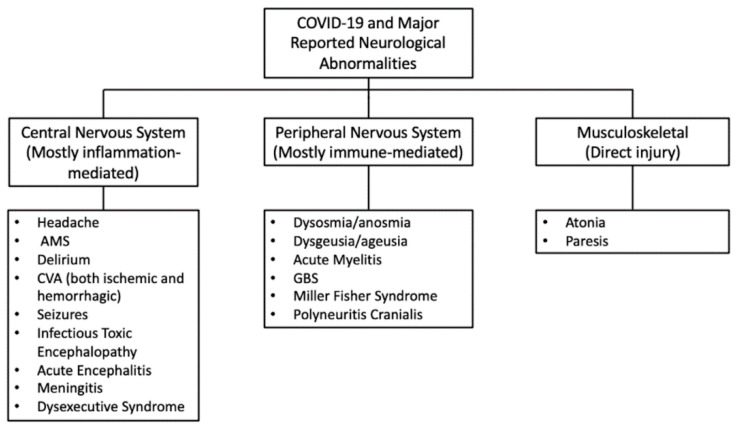
Major neurological abnormalities reported in COVID-19 [87]; AMS—Altered mental status; CVA—Cerebrovascular accidents; GBS—Guillain-Barre syndrome.

Common neurological complications related to COVID-19 are presented in Figure 6 below.

Most of the data of neurological complications in COVID-19 infection come from several observational studies from China, France, the UK and Italy. Summarized data are presented in Table 1**.**

## 8. The Spectrum of Clinical Symptoms of Neuro-COVID Can Be Divided in Early and Late Symptoms

Early neurological symptoms include the loss of sense of smell and taste and also body aches, headache and myalgia.

Anosmia/ageusia, fever, and myalgia are considered the strongest independent predictors of positive SARS-CoV-2 assays. Rapidly developing neurological complications manifesting usually at the beginning of the infection include dizziness, headache, ataxia, seizures. Three recent case reports describe the development of acute parkinsonism following coronavirus disease 2019 (COVID-19) and this has generated concern on the possible induction of PD by SARS-CoV-2 [105]. This case and previous evidence of possible link between coronaviruses and PD is alarming the scientific community of a potential role of COVID-19 in the potential emergence of Parkinsonism as a third wave of consequences of the SARS-CoV-2 pandemic [106,107].

COVID-19-related neurological damage is often recognized late in the course of the disease and its predictive significance remains unclear. Dyspnea remains the only strongly predictive symptom for both moderate and severe disease that can be useful in guiding clinical management decisions early in the course of illness [108].

Late neurological manifestations are impaired mental status, acute cerebrovascular accidents such as ischemic and hemorrhagic stroke, and conditions associated with neural demyelination. Other complications associated with SARS-CoV-2 are meningitis, encephalomyelitis, encephalitis, acute necrotizing hemorrhagic encephalopathy, and Guillain–Barré syndrome (GBS) [109] (Table 1).

### Some Controversies about the Clinical Manifestations of Neuro-COVID

During the ongoing COVID-19 pandemic, patients have experienced different and less frequent symptoms including headache (6.5%), dizziness (9.4%), nausea (10.1%), vomiting (3.6%), abdominal pain (2.2%), and shock (8.7%), in addition to the characteristic symptoms such as fever (98.6%), fatigue (69.6%), dry cough (59.4%), anorexia (39.9%) myalgia (34.8%), dyspnea (31.2%), arrhythmia (16.7%), and acute cardiac injury (7.2%) [90]. Some patients have also reported difficulty breathing as well as gait alterations [68,110] (Table 1).

Although the involvement of both the CNS and PNS is now well documented, the percentage of patients suffering from these complications varies widely across different studies, assessment time-points as well as geographical location [86]. For example, the incidence of neurological symptoms in patients in France was 14% at the time of admission in hospitals and 69% after the discontinuation/completion of the therapeutic course [93]. Various neurological symptoms [68] were reported for 36.4% of the patients in Wuhan, China. CNS complications include dizziness (16.8%), headache (13.1%), altered consciousness (7.5%), stroke (2.8%), ataxia and seizures (0.5%); reported complications related to autonomic nervous system were impaired sense of smell (5.6%) and taste (5.1%) as well as visual disturbances (1.4%). Musculoskeletal complications accounted for 10.7% of all cases with neurological complications, and neuralgia was reported by 2.3% of patients (Table 1).

Data generated in the UK indicate that some patients with COVID-19 may suffer from encephalopathies accompanied by delirium/psychosis while others develop inflammatory CNS syndromes including encephalitis, acute disseminated encephalomyelitis and peripheral neurological disorders like GBS, while a few have life-threatening ischemic strokes [88].

Varatharaj et al. [96] reported neurological/psychiatric illnesses in 125 patients with COVID-19 for 3 weeks. One third of the patients (31%) had altered mental status, including 13% with encephalopathy and 18% with neuropsychiatric diagnosis entailing psychosis (8%), neuro-cognitive syndrome (5%), and affective disorder (3%). The authors state that 62% of the observed patients suffered cerebrovascular events, 74% of which were ischemic strokes, 12% intracerebral hemorrhages, and 1% CNS vasculitis.

Although some authors consider that established neurological complications are most often associated with a severe clinical picture of the viral infection or with concomitant diabetes or hypertension [68,80] (Table 1) contradictory data have been generated in this regard. In patients with moderate symptoms of viral infection or even during recovery, COVID-19 may cause fatal brain and neurological damage of immune–inflammatory origin that leads to destructive changes of the myelin sheath of neurons in the brain and spinal cord leading to paralysis [84].

CNS damage in the absence of lung symptoms has also been reported [81] in young patients (below 50 years of age) developing hemiplegia and impaired consciousness without other symptoms at the same time, and brain MRIs have demonstrated evidences of cerebral artery occlusion.

It is also of interest, in this regard, that the findings of a recent meta-analysis reporting abnormal EEG findings in patients with COVID-19-related encephalopathies included altered mental status, seizure-like events, and cardiac arrest. Abnormal EEG findings were observed in 88.0% of the 543 patients and were sub-classified into three groups including background abnormalities, periodic and rhythmic EEG patterns, and epileptiform changes. It was also shown that approximately a third of all findings consisted of frontal EEG patterns. In studies that utilized continuous EEG, 96.8% of the 251 patients were reported to have abnormalities compared to 85.0% of the patients who did not undergo continuous EEG. Frontal findings were therefore frequent during Neuro-COVID and can be proposed as a marker for its development [111]. These data highlight the potential utility of using EEG for patients with COVID-19 infection exhibiting signs of CNS involvement.

Many authors suggest that the neurotoxic effects of coronaviruses leading to life-threatening neurological conditions are not necessarily linked to the manifestation of severe respiratory symptoms [65,66,69].

As regards peripheral neurological symptoms, a recent study showed that the most common peripheral COVID-19-related symptoms of infection in China included the loss of the sense of taste and smell (5%), visual disturbances (1.4%) and neuralgia (2.3%) [68] (Table 1).

In Italy [97], different degrees of ageusia and anosmia have been reported in 10.2% and 5% of the patients, respectively, while 18.6% of patients complained of both. These symptoms were more common in young patients and in women.

Other studies conducted in 12 European clinics showed that cases of mild to moderate disease were accompanied by olfactory (85.6%) and taste (88%) alterations with a high degree of correlation between them and also predominant in females [101]. For most patients, the recovery of sensitivity took several weeks and 44% of the patients recovered earlier (Table 1).

Musculoskeletal injuries are 10.7% prevalent, mainly in patients with liver and kidney problems, according to Mao et al. [68]. Another study, involving 1099 patients from 550 hospitals in China, reported the occurrence of myalgia (14.9%). The severity of the observed symptoms correlated with the severity of the disease [112]. Rhabdomyolysis was observed in 0.2% of cases (Table 1).

Based on the available information, Sheraton et al. (2020) [87] suggested that CNS symptoms mainly occur in connection with virus-induced inflammation. The PNS complications are secondary to immune-mediated processes, whereas musculoskeletal involvements are the result of the direct damaging effects of the virus [87].

Cerebrovascular accidents usually occur in severe cases of the disease, affecting predominantly elderly patients with cardiovascular and cerebrovascular comorbidities and diabetes.

However, new data indicate that coronavirus-related ischemic strokes may not necessarily be age-related. In New York, five coronavirus patients in their 30s and 40s, most of whom had no past medical history, experienced life-threatening ischemic strokes. These cases may be multifactorial, on the one hand being related to the affinity of SARS-CoV-2 to endothelial cells, which leads to endothelial damage and the formation of blood clots even in individuals with no history or risk of coagulation disorders [103], and on the other to the formation of antiphospholipid (anti-PL) autoantibodies [36].

We already discussed that cerebrovascular events may be due to the presence of antiphospholipid (anti-PL) autoantibodies and that the presence of these autoantibodies should be tested in patients with coagulopathy and the elevation of d-dimer and lupus-like anticoagulant [36], which may be secondary to the upregulated activation of B lymphocytes [113].

## 9. Post-COVID-19 Neurological Recovery

Due to the recent onset of the ongoing pandemic, it is still difficult to assess the risk of short-term or long-term complications in recovering patients. At present, there are more than 43 million people who have been infected with SARS-CoV-2 and many of those who recovered carry the burden of cognitive and neurological sequelae [112,113]. All this is expected to affect their quality of life, ability to work and everyday activity. To date, the observed complications following COVID-19 recovery include demyelination and acute myelitis affecting the spinal cord [114] or cerebral edema accompanied by delirium. A study involving 29 patients categorized post-COVID-19 neurological complications into five main groups [88]. Some altered psychological parameters related to viral diseases were also reported as stress, depression, anxiety, and psychological abnormalities [115,116].

It is worth recalling in this setting the Spanish flu pandemic of 1918 and the fact that more than 1 million people were diagnosed with encephalitis lethargica or “the sleepy sickness“ between 1917 and 1930. Damages, as a result of brain swelling, made many patients disabled by producing constant sleepiness and severe neurodegeneration [117].

That multiple neuropsychiatric symptoms may be expected in the recovery phase of COVID-19 infection and especially those suffering from Neuro-COVID, which is in accordance with data from the SARS-CoV epidemic. It has been reported [118] that up to 42.5% of SARS survivors developed long-lasting psychiatric morbidity conditions that persisted at 4 years follow up. Conditions included mainly post-traumatic stress disorders (54.5%), depression (39%), somatoform pain disorder (36.4%), panic disorder (32.5%) and obsessive–compulsive disorder (15.6%) [118].

The full assessment of the long-term risk of neurological complications will be very much needed once the COVID-19 pandemic is over so to weigh the delayed effects on the brain. There is concern that the coronavirus could bring an epidemic of “brain damage linked to the pandemic” because some brain-damage issues may not manifest for some time after patients have left the hospital [119].

In particular, Beaucham and colleagues fear the development of SARS-CoV2-induced Parkinsonism, and suggest monitoring of recovering individuals for potential long-term consequences, which may include neurodegenerative impairments. [107].

We also previously discussed the risk posed by the eventual long-lasting persistence of anti-PL autoantibodies in people with COVID-19 that eventually develop secondary anti-phospholipid antibody syndrome (APS) [36]. Cases of cerebrovascular events have been reported in patients positive for anti-PL autoantibodies and the kinetics of the disappearance of these autoantibodies remains to be studied. Patients that are positive for anti-PL autoantibodies will require careful laboratory monitoring and eventual anticoagulant treatment until the persistent disappearance of the autoantibodies.

Another aspect of the COVID-19 sequelae that has to date probably been underestimated is the occurrence of chronic fatigue. Since the beginning of the pandemic, the international guidelines on the management of the infection were focused on the acute phase of the disease and the relatively severe complications and sequelae. However, several patients that recovered from COVID-19 infection are increasingly reporting chronic fatigue regardless of whether or not they had neurological complications during the course of the disease [120]. Carfi et al. report that as many as 87.4% of patients assessed approximately 60 days after discharged report at least one persisting symptom, particularly fatigue and dyspnea [120].

Several follow-up studies on patients infected with SARS-CoV also suggest that coronavirus can cause long-term fatigue. In a study conducted on 22 SARS survivors, it was found that they all remained unable to return to work and to their pre-SARS daily life in a period of time ranging between 13 and 36 months after declared infection free [121]. In another study assessing 233 SARS survivors in China, 40% were reported to have suffered chronic fatigue 4 years after recovery [115].

A limiting factor to assessing the severity of chronic fatigue is that there are no data for the level of fatigue before the coronavirus disease and especially since there is no biomarker or any other approach to objectively assess fatigue. The evaluation is based solely on the information provided by the patients which complicates the efficient management of this symptom.

Based on current knowledge and on the reported experience with SARS-CoV we hypothesize that the neuro-behavioral sequelae of SARS-CoV-2 will necessitate a close interaction between primary care, emergency medicine, in-patient treatment, as well as psychological and psychiatry interventions at specific phases and stages. The use of psychotherapy may also be anticipated as it has been suggested that it may lead to measurable neuroimaging changes associated with functional improvement. The beneficial effects of a psychosocial intervention on the function of the immune system with changes persisting for at least 6 months following treatment have also been demonstrated [122]. This may be of particular relevance given the immune pathogenesis of severe cases of COVID-19 that may cause subtle but long lasting derangement in the homeostasis of the immune system.

Along this line of reasoning, Pallanti et al. [123] suggested the utility of the stratification of patients recovered from COVID-19 based on an inflammatory panel that may help design appropriate therapeutic and rehabilitative programs entailing a reduction of stigma associated with mental health sequelae.

## 10. CAVEAT for the Neuropsychological Welfare of Newborns during the COVID-19 Pandemic

A point that has been anticipated in a recent review and that deserves further attention as it risks to be underestimated is the potential impact that both symptomatic or asymptomatic maternal infections may have on the offspring [123]. There are several factors that may jeopardize a successful physiological pregnancy during COVID-19 infection. This may pose a risk in the early phases of neurodevelopment in children because the exposure of the mother to inflammation during pregnancy is associated with the later development of neuropsychiatric disorders in human offspring [124]. This is consistent with the known role played by proinflammatory events in the pathogenesis of mood disorders [125], possibly via the release of inflammatory mediators such as certain cytokines that impact neurodevelopment [75,126,127,128].

We anticipate that the offspring of mothers infected with SARS-CoV or SARS-CoV-2 will require continuous monitoring for neuropsychiatric, neuroimmune and inflammatory status in longitudinal studies to better understand the pathophysiology and to allow for early intervention.

## 11. Rehabilitation after COVID-19

Italy was among the most severely affected countries with very high infection incidence as well as a very high number of severe cases. The analysis of the data gathered in Italy indicates that COVID-19 management should not be concluded with the completion of the therapeutic plan and negative assays but it should also imply a rehabilitation plan for the reconvalescent patients [80].

Neurological complications in patients with viral infection, which also increase the risk of a more severe course of the disease, are a prerequisite for harder and slower recovery after the discontinuation of the artificial ventilation used in acute respiratory distress syndrome. The longer and more severe convalescence period is also related to the need for better rehabilitation.

Some of the COVID-19 survivors may need a long rehabilitation period, both physical rehabilitation such as exercise and mental/cognitive rehabilitation.

## 12. The Need of Developing Accurate “Theranostics” for Early Diagnosis and Treatment of COVID-19 Patients at Risk for Developing Acute or Chronic “Neuro-COVID”

Once that the involvement and the relevance of neurological complications during COVID-19 infection has been established, it will be important to evaluate potential biomarkers that may afford the early identification of patients at risk of Neuro-COVID as well as designing tailored therapeutic approaches for its prevention. Along this line of research, for example, we and others have shown that the mTOR (the mammalian target of rapamycin) pathway, which regulates multiple processes of cell growth and metabolism, also implicated in response to viral infection, is hyperactivated in response to SARS-Cov-2 infection [15,129,130].

The hyperactivated mTOR pathway may represent a potential pharmacological target for COVID-19 management. [15,129,130].

Although mTOR hyperactivation in CNS during COVID-19 remains to be demonstrated, it is of interest to recall that the mTOR pathway has been implicated in several neurological diseases characterized by different extent of inflammation and degeneration such as multiple sclerosis [131], Alzheimer’s disease [132] and HIV-associated neurocognitive decline [133].

We believe that evaluating the association between Neuro-COVID and its severity and mTOR and its upstream pathways PI3K and Akt activation both in PNS and CNS may offer important pathogenetic information and guide toward tailored therapeutic approaches based on inhibitors of either mTOR or the upstream pathways.

As indicated, other potential players in the pathogenesis of Neuro-COVID are the pro-inflammatory cytokines and chemokines that are triggered by SARS-CoV2 infection. It is known that pro-inflammatory cytokines contribute to the pathogenesis of several neurological and psychiatric disorders including Multiple sclerosis (MS), Alzheimer’s disease and major depressive disorders [75,127,134,135]. Identifying one or more of the key cytokines triggering the neurological manifestations of SARS-CoV-2 would allow for their pharmacological targeting to counteract the onset and progression of neuropathology.

In this context, we hypothesized that a relevant role in the pathophysiology of Neuro-COVID may be played by brain-derived neurotophic factor (BDNF), which is the member of a family of neuronal growth factors that also include nerve growth factor (NGF), Neurotrophin-3 (NT-3), and NT-4/5. BDNF regulates neuronal maintenance, neuronal survival, plasticity, and neurotransmitter regulation. Patients with psychiatric and neurodegenerative disorders often have reduced BDNF concentrations in their bloods and brains that may be secondary to the chronic inflammatory state of the brain in certain disorders [136], since BDNF is downstream the ACE2-Mas axis [137].

It has therefore been hypothesized that the interaction of SARS-CoV-2 With ACE-2 may leads to the subsequent impairment of the downstream Mas/BDNF axis with the consequential reduction of BDNF and this may be centrally implicated in the pathogenesis of Neuro-COVID.

The modification of BDNF in COVID-19 and especially in Neuro-COVID remains to be studied. To date, one preliminary study conducted in 16 patients has found significantly lower levels of serum BDNF in patients with severe or moderate disease as compared to patients with mild disease. The significance, if any, of this finding on the role of BDNF in the pathogenesis of COVID-19 infection and in particular of Neuro-COVID, are a clearly interesting area of research [138].

## 13. Conclusions

It is now firmly established on the ground of clinical and pathological evidence that COVID-19 is not a purely respiratory infection but rather a multi-organ disease. This is mainly related to the capacity of the virus to bind the widely expressed ACE2 receptor along with its use of the cleavage protease (furin) which can be found in almost all human tissues. Furthermore, SARS-CoV-2 has strong affinity to the endothelium cells and results in endothelial damage, increased coagulation and disseminated micro-thrombosis.

Several studies are also convergently depicting the frequent involvement of CNS and PNS during the course of COVID-19 leading to specific manifestation of the disease termed Neuro-COVID. Nervous system involvement occurs through direct or indirect mechanisms that provoke a broad spectrum of clinical symptoms including the failure of the regulation of respiratory function. We presently described the most common symptoms related to Neuro-COVID and proposed direct and indirect pathogenetic mechanisms of viral damage. We underscore that the understanding of the mechanisms of CNS and PNS damage is becoming essential for the development of approaches for early diagnosis of individuals at risk for developing Neuro-COVID as well as for the discovery of novel tailored therapeutic approaches. We hypothesized that the reported activation of the mTOR pathway and the upregulated production of pro-inflammatory cytokines in COVID-19 will represent novel valuable theranostics for the prevention and treatment of Neuro-COVID. In particular, emerging evidence suggests that alterations of both systemic and CNS levels of BDNF may occur in COVID-19 and that may be related to CNS symptoms and BDNF. We also suggest that particular attention should be paid to the early neurological and psychiatric development of newborns from mothers with COVID-19 infection for the known effects of immune-inflammation during pregnancy. Finally, we warn for the need for the accurate follow-up of patients with Neuro-COVID during the recovery phase to prevent long-term cognitive and neurological consequences. On the basis of current knowledge and on the reported experience with SARS-CoV, we emphasize that the neurobehavioral sequelae of SARS-CoV-2 will necessitate a close interaction between primary care, emergency medicine, in-patient treatment and mental health care with tailored interventions at specific phases and stages.

## Figures and Tables

**Table 1:** Clinical experience with COVID-19 in different countries.

Country	COVID-19 Cases (*n*)	Main Neurological Complications	Authors
China Wuhan Retrospective Case Series	*n* = 214	Dizziness (16.8%), headache (13.1%), altered consciousness (7.5%), stroke (2.8%), ataxia and seizures (0.5%), hypogeusia (5.6%), hyposmia (5.1%), neuralgia 2.3% skeletal muscle injury (10.7%), visual disturbances (1.4%)	Mao et al., 2020 [68]
China Zhejiang Retrospective Case series	*n* = 62	Headache (34%), myalgia or fatigue (52%)	Xu et al., 2020 [89]
China Wuhan Retrospective Case Series	*n* = 138	Headache (6.5%), dizziness (9.4%), nausea (10.1%), vomiting (3.6%), abdominal pain (2.2%), shock (8.7%) fatigue (69.6%), anorexia (39.9), myalgia (34.8%)	Wang et al., 2020 [90]
China Retrospective Study	*n* = 219	Ischemic stroke (4.6%), cerebral venous sinus thrombosis (0.5%), hemorrhagic stroke (0.5%)	Li Y. et al., 2020 [67]
China, Hubei Sichuan Chongqing Retrospective Study	*n* = 304	Encephalopathy (2.6%), systemic or direct brain insults (27%)	Lu et al., 2020 [91]
China Meta-Analysis (10 Publications)	*n* = 1994	Myalgia or fatigue (35.8%), headache or dizziness (12.1%), nausea and vomiting (3.9%)	Li et al., 2020 [92]
France Strasbourg, Observational Case Series	*n* = 58	Confusion (65%), agitation (69%), diffuse corticospinal tract signs (67%), dysexecutive syndrome at discharge (33%), dizziness (16.8%), headache (13.1%)	Helms et al., 2020 [93]
France Retrospective Observational Study	*n* = 37	Alteration of consciousness (73%), pathological wakefulness after sedation (41%), confusion (32%), agitation (19%), seizures (14%), headache (11%)	Kremer et al., 2020 [94]
France Case Report	*n* = 1	Guillain–Barré syndrome	Camdessanche et al., 2020 [95]
UK Cross-Specialty Surveillance Study	*n* = 125	Cerebrovascular event (62%): ischemic strokes (74%), intracerebral hemorrhage (12%), central nervous system vasculitis (1%). Altered mental status (31%): unspecified encephalopathy (13%) and encephalitis (18%).	Varatharaj et al., 2020 [96]
UK London Retrospective Descriptive Study	*n* = 43	Encephalopathies (23%) with delirium/psychosis Inflammatory CNS syndromes (28%) Ischemic strokes (18.6%) Peripheral neurological disorders (18.6): Guillain–Barré syndrome (16.3), brachial plexopathy (2.3%), Central disorders (11.6%)	Paterson et al., 2020 [88]
Italy Milan Cross-Sectional Study	*n* = 59	Gustatory/olfactory disorders (33.9%), headache (3.4%), asthenia (1.7%)	Giacomelli et al., 2020 [97]
Italy, Milan Single-Center Retrospective Study	*n* = 388	Ischemic stroke (2.5%)	Lodigiani et al., 2020 [98]
Italy	*n* = 5	Guillain–Barré syndrome	Toscano et al., 2020 [99]
Italy Milan Case Series	*n* = 4	Subacute encephalopathy, neurological signs of agitation and spatial disorientation	Anzalone et al., 2020 [100]
EU Belgium, France, Italy, Spain, Switzerland Cross-Sectional Study	*n* = 417	Olfactory dysfunctions (85.6%), gustatory dysfunctions (82%)	Lechien et al., 2020 [101]
Netherlands Multicenter Retrospective Study	*n* = 180	Ischemic stroke (3.7%)	Klok et al., 2020 [102]
USA Case Series	*n* = 5	Hemiplegia (100%), reduced consciousness (80%), dysarthria (60%), dysphasia (20%), sensory deficit (40%)	Oxley et al., 2020 [103]
USA Case Study	*n* = 1	Altered mental status; hemorrhagic necrotizing encephalopathy	Poyiadji et al., 2020 [104]
